# Maximization through optimization? On the relationship between hybrid performance and parental genetic distance

**DOI:** 10.1007/s00122-023-04436-5

**Published:** 2023-08-12

**Authors:** Tobias Würschum, Xintian Zhu, Yusheng Zhao, Yong Jiang, Jochen C. Reif, Hans Peter Maurer

**Affiliations:** 1 Institute of Plant Breeding, Seed Science and Population Genetics, University of Hohenheim, 70599 Stuttgart, Germany; 2 Leibniz Institute of Plant Genetics and Crop Plant Research (IPK), 06466 Stadt Seeland, Germany; 3 State Plant Breeding Institute, University of Hohenheim, 70599 Stuttgart, Germany

## Abstract

Heterosis is the improved performance of hybrids compared with their parental components and is widely exploited in agriculture. According to quantitative genetic theory, genetic distance between parents at heterotic quantitative trait loci is required for heterosis, but how heterosis varies with genetic distance has remained elusive, despite intensive research on the topic. Experimental studies have often found a positive association between heterosis and genetic distance that, however, varied in strength. Most importantly, it has remained unclear whether heterosis increases continuously with genetic distance or whether there is an optimum genetic distance after which heterosis declines again. Here, we revisit the relationship between heterosis and genetic distance and provide perspectives on how to maximize heterosis and hybrid performance in breeding, as well as the consequences for the design of heterotic groups and the utilization of more exotic material and genetic resources.

## Heterosis

Heterosis, or hybrid vigor, is a phenomenon that has been recognized for over a century and refers to the increased performance of crosses compared to their parental components (Lippman and Zamir 2006). It is often expressed as midparent heterosis, which is the deviation of the hybrid performance from the mean of its parents (Schnable and Springer 2013). Heterosis is exploited for agriculture in the form of hybrid breeding. The most prominent crop example is maize, where the introduction of hybrid varieties in the 1930s has substantially contributed to the impressive increase in yield (Lamkey and Edwards 1999). Hybrid breeding has been particularly successful for outcrossing species, besides maize for example rye and sugar beet, but in recent years has also received renewed and increasing interest for selfing crops like rice, wheat, and barley (Longin et al. 2012; Selva et al. 2020).

The genetic basis of heterosis has intrigued scientists and breeders for many decades and has inspired theorizing and experimentation. Despite these efforts, the genetic and molecular causes of heterosis are still not fully understood. Nevertheless, several competing but mutually nonexclusive genetic models to explain heterotic effects have been proposed and experimentally validated (Stuber et al. 1992; Melchinger et al. 2007; Jiang et al. 2017; Yang et al. 2017). The dominance model proposes that deleterious alleles present in the parents at many loci are complemented in the hybrid by dominant alleles from the other parent, resulting in phenotypic superiority over both parents. The overdominance hypothesis holds that heterozygosity at individual loci leads to superior phenotypic performance relative to that of either homozygous condition. Both hypotheses are based on interactions between alleles at single loci, whereas the epistasis hypothesis predicts that heterosis results from favorable interactions between loci. According to quantitative genetic theory, heterosis depends on the genetic distance between the parents. Genetic distance refers to the squared difference in allele frequencies between parents, taking into account all quantitative trait loci (QTL) that contribute to heterosis (Falconer and Mackay 1996; Jiang et al. 2017). In other words, none of these hypotheses can take effect if there are no genetic differences between the parents of a hybrid.

### Inbreeding and outbreeding depression

The converse of heterosis is inbreeding depression which refers to the decrease in vigor when close relatives are mated or selfings are created and is caused by deleterious recessive alleles becoming homozygous. It is routinely experienced in hybrid breeding of outcrossing crops when inbred lines are generated, which are homozygous lines and, thus, represent fixed gametes that can be used indefinitely to generate the desired hybrids. However, a reduction in fitness and vigor as for inbreeding can also occur by cross-mating of different populations, a phenomenon termed outbreeding depression (Lynch 1991). Typically, alleles of different loci do not act alone in an organism but interact and therefore co-evolve so that their products act harmoniously with each other. This ensures a high level of fitness within a population, but if different combinations of alleles evolve in different populations, these may perform poorly when mixed through cross-mating. The Bateson–Dobzhansky–Muller model of genetic incompatibility and speciation explains how simple epistatic interactions can result in detrimental effects in hybrids, up to hybrid sterility and death, even though both parental components are vigorous and fertile (Orr 1996). This model is generally studied in the context of the process of species formation, but genetic incompatibilities are also well known within species (Seidel et al. 2008; Corbett-Detig et al. 2013; Cutter 2012).

This incompatibility within species can be illustrated by a phenomenon described as hybrid necrosis that can be classified as an extreme form of outbreeding depression. Hybrid necrosis was first documented more than a century ago but only recently has been genetically and molecularly characterized and shown to often be a genetic autoimmunity syndrome that can occur in both interspecific but also in intraspecific crosses (Bomblies and Weigel 2007; Li and Weigel 2021). Plants are exposed to a range of pathogens and have evolved sophisticated defense mechanisms as a result of this long-term evolutionary conflict. Consequently, the loci involved in this response are often highly variable resulting in a vast diversity, which increases the probability that separately evolved alleles or genes are not co-adapted. If such non-co-adapted genetic variants are brought together in a hybrid, the highly regulated and finely tuned pathways underlying plant immunity may fail, resulting in an erroneous activation of the immune response, like tissue necrosis and cell death, and thus detrimental effects on the hybrid. The hybrid necrosis examples studied so far all show simple genetics, with sometimes just one but mostly two causal loci that often encode immune receptors, and the effects being the consequence of intra- or intergenic epistasis. As such, they conform to the tenets of the Bateson–Dobzhansky–Muller model. A large systematic study on intraspecific hybrid necrosis in *Arabidopsis thaliana* revealed that about 2% of the crosses resulted in hybrid necrosis, but this analysis was limited to cases with strong morphological effect (Chae et al. 2014). Chae et al. (2014) also reported that the genetic distance, which in Arabidopsis is associated with geographic distance, is not a good predictor for hybrid incompatibility. This is likely due to the fact that only few loci are involved and the genetic changes are single events that can occur in any population, independent of the genetic distance to any other population. On the other hand, it is interesting to note that the hybrid necrosis activity of a certain allele appears to have been acquired stepwise, and consequently, more such incompatibilities may arise with increasing divergence. Notably, the severity of hybrid necrosis effects shows a wide range, from very mild defects that only become apparent in a subset of the hybrid progeny to hybrids that die shortly after germination (Bomblies and Weigel 2007). Thus, it is likely that milder forms go unnoticed in studies on hybrid necrosis, as they may not result in the typical symptoms, but nevertheless cause a reduction in hybrid performance and heterosis.

Another interesting observation is that alleles that increase disease resistance in crops and that have consequently been actively selected were found to also be tied to autoimmune risk. A well-studied example comes from tomato, where the introgressed fungal resistance gene from a wild relative results in autonecrosis due to interaction with alleles at an unlinked locus (Krüger et al. 2002). In a wheat example, hybrid necrosis is controlled by the interaction of two dominant genes, *Ne1* and *Ne2*, both characterized by multiple alleles that despite their negative effect have been maintained and are widespread. Genetic work suggested *Ne2* to be identical to the leaf rust resistance locus *Lr13*, a previously important and widely used resistance source (Zhang et al. 2016). Thus, the intensive selection for various disease resistances in breeding programs may be a factor that increases incompatibilities between separated gene pools in crops.

Though autoimmunity may be the most common cause for hybrid necrosis, there are also other possible physiological mechanisms as described by Bomblies and Weigel (2007). These include episomal viral infection which is caused by viral sequences present in the parental genomes that are activated in the hybrid. This is reminiscent of the hybrid dysgenesis phenomenon in Drosophila that results from activation of transposons present in one parent but not in the other, and in general, intragenomic conflicts have been suggested to contribute to a reduced hybrid fitness or fertility. Disturbed hormone signaling or function has also been implicated in cases of hybrid necrosis and another example of hybrid necrosis may be linked to selection for copper tolerance and may thus represent the consequence of adaptation to different environmental conditions (MacNair and Christie 1983). In another case, tight linkage was found between genes causing hybrid necrosis and major genes involved in the adaptation to different pollinators, again illustrating the effects of local environmental adaptation on genetic incompatibilities (Li et al. 2023). Thus, depending on the divergence of the parental components of a cross, different phenomena act on the fitness of generated hybrids, which has consequences for the design and the organization of hybrid breeding programs.

### Hybrid breeding and heterotic groups

The relationship between genetic divergence and hybrid performance was initially unclear and early hybrid breeding in maize was mainly focused on improving the poor agronomic performance of the first-generation inbred lines by making crosses among them (Tracy and Chandler 2006). As a result of this necessity, many of the second- and third-generation inbred lines were derived from crosses between parents from what are now considered opposite heterotic groups. However, based on the experiences in early hybrid maize breeding, it also became evident that a higher heterosis and thus a higher hybrid performance could be achieved with genetically divergent parental components. Efforts were then made to organize breeding programs accordingly and form two groups of lines that were to be kept separate, but interestingly, the assignment of lines to the groups was mostly random and did not follow their genetic origin. Our current concept of heterotic groups and patterns only developed later, probably crystallizing in the late 1960s and early 1970s and becoming more widely recognized in the 1970s and 1980s (Hallauer 1999; Tracy and Chandler 2006). Following the definition of Melchinger and Gumber (1998), a heterotic group “denotes a group of related or unrelated genotypes from the same or different populations, which display similar combining ability and heterotic response when crossed with genotypes from other genetically distinct germplasm groups”, and a genetic pattern describes a specific pair of heterotic groups showing a high level of heterosis. Heterotic groups thus provide a systematic way to exploit the genetic divergence between hybrid components. In general, the groups forming a heterotic pattern will show a certain genetic distance and are developed by reciprocal recurrent selection, which further contributes to their genetic divergence (Gerke et al. 2015; Rembe et al. 2019). In this context it is interesting to note that the divergence between the current US maize heterotic groups stiff stalk (SS) and non-SS did not exist in the original germplasm, owing to the history and the arbitrary grouping mentioned above, but was created by breeding, as reviewed by Tracy and Chandler (2006; Duvick et al. 2004).

### Studies on the relationship between heterosis and genetic distance

**Fig. 1:**
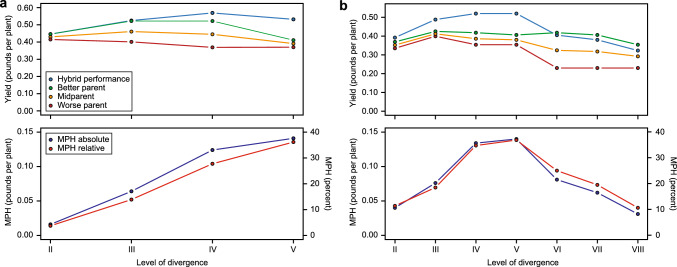
Visualization of the results from maize populations by Moll and coworkers. Average yield and midparent heterosis (MPH) for different levels of divergence between the parental populations shown for **a** Moll et al. (1962) and **b** Moll et al. (1965). The levels of divergence (II—VIII) are according to the original studies and are based on geographic distance and ancestral relationship

The initial observations on the association between heterosis and genetic distance have stimulated more systematic studies on the topic. Especially the work from Moll et al. from 1962 and 1965, with two contrasting results, has formed the basis for controversial discussions in the plant breeding field in the past decades. As molecular markers were not available at the time, geographic distance and the degree of ancestral relationship were used to classify different degrees of genetic divergence between maize populations. In the first study from 1962, Moll et al. used six varieties from three geographical regions, southeastern USA, midwestern USA and Puerto Rico, and observed the highest grain yield heterosis in the crosses with the presumed greatest genetic divergence of the parental varieties (Fig. 1). In a similar study, Moll et al. (1965) used a higher level of parental diversity by including two varieties from southern Mexico and postulated eight levels of divergence. Now, however, they observed a decrease in heterosis in the hybrids from the widest crosses. Notably, the two Mexican populations also performed poorly in their native environment. Thus, from the two studies of Moll and coworkers, it remained unclear whether heterosis increases continuously with genetic distance between the hybrid parental components or whether there is an optimum after which heterosis decreases again.

With the advent of molecular markers, studies became possible that directly measure genome-wide genetic distance as a proxy for the genetic distance at heterotic loci, instead of the indirect measures used by Moll et al. (1962, 1965). Reif et al. (2003), for example, assessed the association of heterosis and genetic distance in tropical maize. They observed an increasing heterosis with genetic distance and no decrease even under their maximum genetic distance, which they attributed to the similar adaptation of the parental component lines and the lack of extremely distant crosses. There are many other studies available that approached this topic in different crops, which, however, often reported only a weak or even no association between heterosis and genetic distance (Jain et al. 1994; Zhang et al. 1995; Xiao et al. 1996; Cheres et al. 2000; Riaz et al. 2001; Betrán et al. 2003). One can of course argue that the material used in these studies was too narrow and the genetic distance simply not large enough to observe this positive association, but as we will see later, there are other effects that need consideration.

Obviously, linkage disequilibrium between markers and heterotic loci is a necessary condition for a correlation between heterozygosity at these marker loci and heterosis, which should generally be given within populations (Charcosset and Essioux 1994). Notably, however, Charcosset and Essioux (1994) showed that as these linkage disequilibria differ between heterotic groups, genetic distances based on neutral marker loci will not be predictive for the performance of between-group hybrids.

So despite its importance for hybrid breeding and the many decades of research on this topic, which more recently was assisted by the availability of molecular markers, the relationship between heterosis and genetic distance remained elusive. The question is whether heterosis is maximized through maximization of the genetic distance between the parental components or rather if heterosis is maximized through an optimization of this genetic distance.

## The snowball and the hump

The Bateson–Dobzhansky–Muller model is generally applied to speciation and the hybrid incompatibility between different species. Now theory predicts that if substitutions differentiating two species accumulate at a constant rate, so linearly, the number of genetic incompatibilities will increase faster than linearly—the snowball effect (Orr 1995; Orr and Turelli 2001). This is because the incompatibility is predicted to result from negative epistatic interactions and the number of two-locus epistatic incompatibilities is expected to increase with the square of the number of substitutions differentiating the species, three-locus epistatic interactions will increase in number with the cube of the number of substitutions between them and higher-order epistatic interactions among more than three loci are expected to increase even faster. There is empirical support for the number of genetic incompatibilities snowballing with the time since species divergence in *Drosophila* and tomato, but also caution in the interpretation based on a re-analysis taking into account the contribution of ancestral polymorphisms to the genetic distance (Matute et al. 2010; Moyle and Nakazato 2010; Städler et al. 2012; Cutter 2012).

**Fig. 2:**
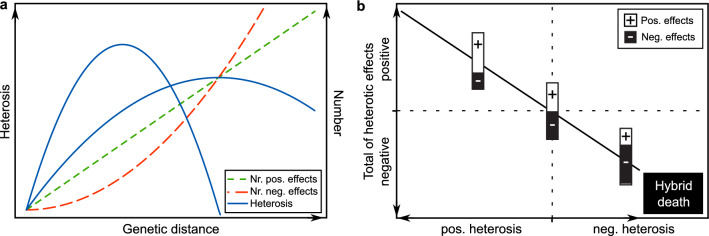
Schematic representation of the possible relationship between heterosis and genetic distance. **a** Illustration of a linear accumulation of the number of positive heterotic effects and a non-linear, snowballing accumulation of the number of negative heterotic effects with genetic distance between the parental components of a hybrid. Their combined effects then results in a hump-shaped relationship between heterosis and genetic distance, with an optimum genetic distance that maximizes heterosis. Here, two of these heterosis curves are shown of which the broader one illustrates the effect of a delayed onset of the accumulation of negative effects and/or smaller negative effects for closer genetic distances. **b** The large spectrum of outcomes of outcrossing is determined by the total of the positive and negative heterotic effects. If this is positive, the heterosis shows varying positive degrees, if, by contrast, the negative heterotic effects outweigh the positive ones, this results in a negative heterosis or outbreeding depression up to hybrid death as observed for severe cases of hybrid necrosis

An interesting study recently suggested that even within a species there is an optimum genetic distance between the parents of a hybrid that maximizes the fitness by balancing the benefits of heterosis and the harm of genetic incompatibility (Wei and Zhang 2018). The authors deduce that dominance more likely results from intragenic interactions between an ancestral allele and a derived allele and is therefore expected to rise linearly with genetic distance of the parents (Fig. 2a). In contrast, the other genetic effects contributing to heterosis, which are overdominance, underdominance as well as positive and negative two-locus epistasis, are more likely to occur between two derived alleles and consequently to increase in numbers in proportion to the squared genetic distance between parents, analogous to the snowball effect. In their model, midparent heterosis depends on two terms: first dominance and second a combined effect arising from overdominance and positive epistasis on the one side and underdominance and negative epistasis on the other side. If both terms are relevant and if the net effect of the former is positive and of the latter is negative, this results in a hump-shaped relationship between midparent heterosis and genetic distance of the parents. Indeed, the authors demonstrate that this model resulted in the best model fit for datasets of yeast, Arabidopsis and mouse, and the optimum mating distance was often similar or slightly greater than the genome-wide nucleotide diversity (*π*) but smaller than the maximum intraspecific genetic distance. Also this model is of course based on assumptions that may not necessarily hold true, like effect sizes being independent of genetic distance and the ratio of positive and negative contributions that enter the second term being constant over genetic distance. In addition, selection in breeding populations is different from natural populations and we must assume a considerable contribution of genetic drift that together would at least partially offset some of the assumptions. Nevertheless, this interesting model provides an explanation and a mathematical framework for an optimal mating distance and also for varying proportions of the different genetic effects over the genetic distance space.

### Theoretical considerations: negative dominance effects

**Fig. 3:**
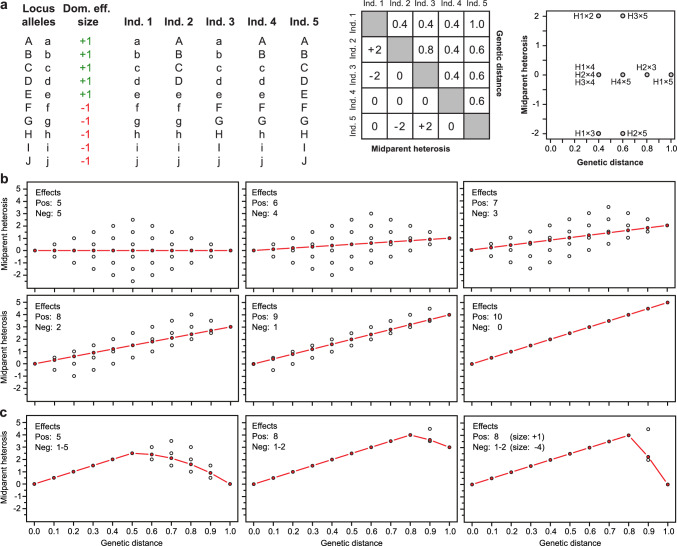
Theoretical considerations regarding negative dominance effects. **a** Assume ten bi-allelic loci, A to J, with the capital letter allele being dominant over the other allele. The degree of dominance is one and for this illustration we simply assume that all loci have an equal effect size, with half of the loci, A to E, having a positive effect of + 1 and the other half, F to J, a negative effect of − 1. Notably, this is equivalent to assuming that positive and negative effects vary in size but both show the same distribution. Next to that is the allelic state of the gametes of five selected homozygous individuals, Ind. 1 to Ind. 5. Shown is then the genetic distance between the five individuals (above diagonal) and the midparent heterosis resulting from loci being heterozygous in the hybrids (below diagonal). Note that Individual 1 has the same genetic distance to Individuals 2, 3 and 4, but the midparent heterosis varies from − 2 to 0 to + 2. The highest heterosis is achieved by hybrids H1 × 2 and H3 × 5 with a genetic distance of 0.4 and 0.6, respectively, and a heterosis of + 2, whereas the hybrid with the largest genetic distance between the parents, H1 × 5, has zero midparent heterosis. **b** Assumed are again the ten loci each having two alleles, for which 1024 allelic combinations and thus parental lines are possible, that are crossed to produce the hybrids. The association between midparent heterosis and genetic distance is shown for six scenarios, with five to zero of the loci having a negative effect. Circles represent possible midparent heterosis values and the red filled circles show the mean for each genetic distance. For the same number of five positive and five negative effects, the maximum positive as well as negative heterosis is achieved for the genetic distance of 0.5, while hybrids that further maximize the genetic distance between their parental components can only have reduced heterosis values. In this setting, the correlation between heterosis and genetic distance is zero. This changes, when we vary the number of negative dominance effects from five to zero. **c** In an alternative scenario the dominance effects are assumed to be positive up to a genetic distance of 0.5, then for a genetic distance of 0.6–1.0 a negative dominance effect is added for every 0.1 distance. The same scenario is also shown assuming positive effects up to a genetic distance of 0.8, once with negative dominance effect sizes of − 1 and then of − 4

As we have just seen, negative heterotic effects that accumulate non-linearly with genetic distance can result in the hump-shaped relationship. However, even if we ignore these other genetic effects for the moment and focus on dominance effects, the expectation of an increasing heterosis with genetic distance is still based on the assumption of dominance effects being positive, so promoting agronomic traits in our favor. This is an understandable expectation but not genetic reality, as dominance effects can also be negative, at least from our perspective and the desired expression of agronomic traits. In that case, heterozygosity at loci with negative dominance effect also increases genetic distance, just as the loci with positive effects, but reduces heterosis (Fig. 3a, b). So in this scenario the hybrid with the highest midparent heterosis is not the hybrid that maximizes the genetic distance, but a hybrid that is only heterozygous at the loci with positive dominance effects and homozygous at the dominance loci with negative effects. If the ratio between positive and negative dominance effects changes, this has a tremendous effect on the association between heterosis and genetic distance. With an equal number and size of positive and negative effects the correlation is zero, whereas with more positive than negative effects we observe a linear positive trend and an increasing correlation.

In natural populations, negative dominance effects are expected to be purged by selection from the populations in which they arise and mainly positive dominance effects will be maintained. In breeding populations, however, the forces of selection are different and local adaptation, different selection targets and strategies, co-selection of linked loci, multi-trait selection as well as genetic drift can result in negative dominance effect loci becoming fixed. Differences in the former may be more pronounced the more separated breeding programs are in time and space and thus, negative dominance effects may occur less often for more closely related material and increase in number the larger the genetic distance between the hybrid components. In such a scenario, midparent heterosis increases with genetic distance up to the point where more negative effects start to accumulate (Fig. 3c). Notably, the strength of the increase as well as the decrease in heterosis with genetic distance not only depends on the number of positive and negative effects but also on the effect sizes. It must also be noted that even if there is a clear non-linear relationship between heterosis and genetic distance in such settings, the linear correlation between them can be zero.

This illustrates that also negative dominance effects can lead to various associations between heterosis and genetic distance that resemble findings from the literature including an optimum genetic distance for heterosis and a decrease for wider crosses. Importantly, the presence and the consequences of negative dominance effects and other genetic effects like negative epistasis are of course not exclusive and it is most likely that both occur and act concurrently.

### Theoretical considerations: epistasis

As for dominance effects, also epistatic effects can be both positive and negative (Boeven et al. 2020). In addition, it must be noted that the midparent heterosis depends not only on the genotype of the hybrid but also on the genotypes of the two parental components, as shown by Jiang et al. (2017). We will demonstrate this by the following example where we consider two bi-allelic loci, A/a and B/b, under the ${F}_{\infty }-$ metric. Assume two parental components, parent P1 having the genotype AABB and parent *P*2 having the genotype aabb, and the resulting hybrid being AaBb. The genotypic value of P1 is ${a}_{1}+{a}_{2}+{aa}_{12}$, that of P2 is ${-a}_{1}-{a}_{2}+{aa}_{12}$, and the genotypic value of the hybrid is ${d}_{1}+{d}_{2}+{dd}_{12}$, where ${a}_{i}$ and ${d}_{i}$ are the additive and dominance effect of locus *i*, respectively, and ${aa}_{ij}$ and ${dd}_{ij}$ refer to the additive-by-additive and dominance-by-dominance effects between locus *i* and *j*, respectively. The midparent (MP) value is (P1 + P2)/2, so here $+{aa}_{12}$ and with this the midparent heterosis MPH = F_1_ − MP becomes ${d}_{1}+{d}_{2}+{dd}_{12}-{aa}_{12}$. Note, that the additive-by-additive epistasis does not contribute to the hybrid performance but does contribute to the midparent heterosis. Now a second pair of parental components is P3 with the genotype AAbb and P4 with the genotype aaBB. Their genotypic values are ${a}_{1}-{a}_{2}-{aa}_{12}$ and $-{a}_{1}+{a}_{2}-{aa}_{12}$, respectively, while the genotype and consequently the genotypic value of the hybrid is the same as for P1 and P2. Now here the midparent value is $-{aa}_{12}$ and the midparent heterosis consequently becomes ${d}_{1}+{d}_{2}+{dd}_{12}+{aa}_{12}$. Note, how the contribution of the additive-by-additive epistasis to the midparent heterosis changes in sign between the two examples. In addition, it is notable that the two hybrids have different midparent heterosis, but the genetic distance between their parental components P1 and P2 or P3 and P4 is the same. So if epistasis is involved and considered, the choice of parental components and the entire design of a hybrid breeding program become more complicated, as this means that there are favorable allelic combinations at two or more loci that need to be brought together in the hybrid.

### Cross-mating can produce a large range of outcomes of hybrid performance

We have now seen that different phenomena act on heterosis and the performance of hybrids and that the sign of the heterotic effects shapes the association with genetic distance. Though the name of outbreeding depression and its effect on hybrid performance suggest a closer relationship with inbreeding depression, it is actually heterosis and outbreeding depression that can be regarded as genetically identical but expressing opposite outcomes of heterotic effects in hybrids. For inbreeding depression, loci change from a heterozygous to a homozygous state, while, by contrast, both heterosis and outbreeding depression result from cross-mating. This typically results in an increase in allelic diversity and heterozygosity in the genome of the hybrid and thus in the associated heterotic effects. Heterosis is then determined by the mixture of positive and negative heterotic effects. We intuitively associate heterotic effects as being positive for heterosis, but the literature also knows examples of hybrids with negative heterosis. So whether cross-mating has a positive effect on hybrid performance, which we then call heterosis, or whether it reduces hybrid performance, then classified as negative heterosis or depending on the context as outbreeding depression, depends on whether the total sum of the heterotic effects and potentially present inbreeding depression is positive or negative (Fig. 2b). Notably, it is not the number of heterotic effects, though in most cases this will be related, but the total of the effects that determines the outcome. This is illustrated with the extreme case of negative heterotic effects in hybrid necrosis, where few loci can have detrimental effects on the hybrid performance as their severity outweighs all possible positive heterotic effects. This spectrum of outcomes of cross-mating, ranging from strong positive heterosis to hybrid death is important for our interpretation of the association between heterosis and genetic distance.

Just as the loci involved in immune response described above, the entire genome is subject to constant change by mutations. This includes neutral mutations in intergenic regions as well as in coding sequences that contribute to genetic distance but have no effect on protein formation or function and thus on trait expression, but it also includes mutations that alter protein function. If this provides a selective advantage, the mutation will be maintained and can become fixed. Notably, as mentioned for the negative dominance effects, fixation of alleles in breeding populations can occur independent from the genetic effect and thus also for more or less deleterious alleles. Within a genetic background, this can put pressure on interacting genes to co-evolve genetic variants and adjust their function in order to not impair fitness. In contrast, there is no selection pressure on these variants to remain compatible with genetic variants arising in separately evolving gene pools. This co-evolution of genetic variants also suggests that it is not necessarily a fixed proportion of polymorphisms between increasingly divergent gene pools that will be negative in a hybrid. In general, however, the larger the genetic distance the longer the separation and with it we can assume not only more diverged gene complexes, but also an increasing incompatibility between them, so larger negative effect sizes as well as an increasing complexity of the non-co-adapted gene networks. Consequently, we hypothesize that there are only few negative heterotic effects in more closely related or co-adapted gene pools and an increasing number and size of negative heterotic effects with increasing genetic distance, though their primary cause will likely vary. If the theory of the hump-shaped distribution holds true and there is an optimum genetic distance that maximizes hybrid performance, then the net effects should become negative after twice the optimum distance. However, how much of this theoretical relationship is covered and consequently, if a decline in heterosis and hybrid performance can be observed, likely depends not only on the experimental setup but also on the crop as well as the evolutionary history of the gene pools of a crop and to what extent they have co-evolved, which can explain the variable observed associations between heterosis and genetic distance.

In this context, it is interesting to note that despite the extensive intercrossing that is common practice in maize, no hybrid necrosis has been reported yet, raising the question if such differences are species-specific or if there is also a general difference between selfing and outcrossing species. This question not only applies to hybrid necrosis but to outbreeding depression in general. In contrast to selfing crops, outcrossing crops suffer strongly from inbreeding because recessive deleterious mutations usually occur in a heterozygous state. However, it remains to be seen what holds true for outbreeding depression, so if outbreeding crops are less or more likely to suffer from it than selfing crops, and consequently, if the natural mating system affects the relationship between hybrid performance and parental genetic distance.

### Establishment of a heterotic genetic distance measure

The above considerations illustrate that unless more dominance effects are positive than negative throughout the genetic distance space, heterosis cannot be expected to linearly increase with genetic distance, even in the absence of epistasis. Boeven et al. (2020), therefore, recently proposed and implemented a heterotic genetic distance measure that allows to incorporate information on the genetic architecture of heterosis. To this end, dominance effects are first estimated in a Bayesian genome-wide approach (Zhao et al. 2015). The heterotic genetic distance is then a weighted function of the Rogers’ distance (${f}_{\mathrm{RD}}$) that incorporates the estimated dominance effects as the weight for each locus. This means that if a locus has no dominance effect, it will not contribute to the heterotic genetic distance measure, while otherwise the weight given to each locus will depend on its sign and effect size. Notably, only positive dominance effects will increase the heterotic genetic distance whereas negative dominance effects will reduce it. Boeven et al. (2020) applied this to a large hybrid wheat data set with 1903 hybrids that strongly differed in their degree of divergence, as crosses within as well as among elite and exotic lines were included. This revealed a strong and monotonic increase in midparent heterosis with heterotic genetic distance of the parental components of the hybrids, while this trend was only weakly expressed for the normal genetic distance. Taken together, this illustrates the importance of considering the genetic architecture of heterosis when assessing the relationship between heterosis and genetic distance.

### The effects of adaptation on hybrid performance and heterosis

The effects of adaptation on studies on hybrid performance and heterosis are twofold. On the one hand, adaptation issues can experimentally confound results, and on the other hand, adaptation pathways and loci are likely a major contributor to the negative heterotic effects of outbreeding depression. Moll et al. (1962) already discussed the possible confounding effects of adaptation, if some varieties and the derived hybrids are not adapted to the test environments. They, however, concluded that in their study, differences in adaptation were small and would not affect the observed association of heterosis and genetic distance. In the follow-up work, the experiment was performed in each region represented by the included populations and the same trend was consistently observed among the four test locations (Moll et al. 1965). Nevertheless, even with such an experimental setup, an effect of adaptation on the results cannot be ruled out.

The relative midparent heterosis will not be affected if the effect of the environment on the hybrid and the non-adapted parental component(s) is proportional. But if, for example, plants do not mature in time due to adaptation issues or are severely affected by diseases to which they are not accustomed, then they cannot realize their true genetic yield potential and the estimated heterosis values can be biased. The direction of the confounding effect of adaptation on heterosis then depends on how the non-adapted parental component(s) and the hybrid are affected relative to each other. If one or both parental components are more severely affected than the hybrid, the midparent value will be reduced and the heterosis will be upwards biased. By contrast, if the hybrid suffers more strongly, this will reduce heterosis. Notably, this confounding effect of adaptation can also be caused by additive genetic effects, if the pleiotropic effect on grain yield or other target trait(s) is not additive, as may for example be the case when harvesting matured and not fully matured plots.

On the other hand, adaptation is a likely cause of negative heterotic effects, as also the non-adapted allele can be dominant. In addition, the molecular pathways underlying adaptation are often finely tuned and thus epistatic incompatibilities can more easily arise if these co-evolved gene complexes are disrupted in an inter-population hybrid, which then has a higher probability of performing poorly. So ideally, experiments on heterosis would be done with only adapted material to exclude the potential confounding effects of adaptation. In practice, however, this will not be possible, as genetic distance will inevitably be associated with geographic distance and genetically more distant material will therefore in most cases also show a different adaptation, but even geographically close material may differ in its adaptation.

### Consequences for hybrid breeding with established heterotic groups

From the above, we have seen that genetic distance is required for heterosis, but heterotic effects can be positive and negative and heterosis depends on maximizing the ratio of the total of the positive contributions relative to the negative ones. So what determines the number of positive and negative heterotic effects in established heterotic groups? Within a population, selection can work against segregating negative effects and can favor positive ones, but hybrid breeding is based on bringing together different populations.

If lines are used, as is typical in hybrid breeding of both selfing as well as outcrossing crops, dominance effects cannot contribute to the per se improvement of each of the groups, as there is no heterozygosity in the lines as the final products. Nevertheless, there may be selection against the unfavorable allele, either in the homozygous state or if lines are developed through recurrent selfing also in the heterozygous state in early generations. Improvement of two heterotic groups in a heterotic pattern, however, occurs through reciprocal recurrent selection, which means that for negative dominance effect loci both heterotic groups should be pushed toward the favorable allele, as otherwise the hybrids between them are heterozygous for such loci which reduces heterosis and hybrid performance. This process may be rather slow due to the small effects of such loci for quantitative traits and the consequently low selection pressure against them. Notably, heterotic loci can be genetically independent from others but can also occur in linkage. If a positive heterotic locus with a small absolute effect is linked to a negative heterotic locus with a large absolute effect, selection may work against the latter and the same haplotype may get fixed in both groups thereby reducing the potentially possible genetic distance but benefitting heterosis and hybrid performance. This linkage between heterotic loci will also affect heterosis in most crops due to the commonly performed multi-trait selection, so that selection against a negative heterotic effect for one trait will reduce not only the genetic distance but potentially also the heterosis for another trait. Nevertheless, negative dominance effects are likely to be reduced over time within an existing heterotic pattern. Scientific studies on heterosis that are based on established heterotic patterns can, as a consequence, be expected to find a rather low proportion of negative heterotic effect loci and mainly those with very small effect sizes are remaining that anyhow cannot be mapped and identified. Thus, for any distant or not, non-co-selected groups of material, there should be more negative heterotic effects than for co-evolved groups. An indication for this comes from the recent study of Boeven et al. (2020) in wheat, where hybrids between elite material, that is characterized by a long history of exchange and intercrossing, showed a lower number of negative dominance and dominance-by-dominance effects than hybrids resulting from crosses between elite and exotic lines.

An important question that arises from these considerations is, if crosses between groups of a heterotic pattern might be advantageous to increase heterosis and hybrid performance. This challenges the central and long-standing paradigm of hybrid breeding that poses that the heterotic groups of an established heterotic pattern should be kept separated. This strict separation has been standard practice in hybrid breeding for decades to maximize heterosis but also as it simplifies hybrid breeding and offers a more favorable ratio of general (GCA) to specific combining ability (SCA) variances. However, just as for other germplasm groups, negative heterotic effects may also be present in the two groups of an established heterotic pattern. If for such loci the different alleles are fixed in the two heterotic groups, there is no way to select against them and the hybrids resulting from crosses between lines of these groups will inevitably be heterozygous and thus carry the burden of these negative heterotic effects. Fixation of the different alleles in the two groups may have existed from the very start in cases where the loci are not polymorphic in either of the founder groups, or may have arisen later through selection on certain genomic regions or simply by random genetic drift. It thus seems justified to assume that such negative heterotic effects exist also in established heterotic groups of adapted elite hybrid breeding material. In addition to reducing heterosis, the fixation of different alleles in the two groups can also further affect the hybrid performance. For loci with additive genetic effect, the hybrids cannot reach the performance of the favorable homozygous state, but only the intermediate value of the two homozygous classes.

This situation can only be broken if the favorable allele is introgressed in the group where it is not present. This might be achieved by introgression of genetically distant material, however, with the potential disadvantage of adaptation issues or outbreeding depression associated with such material. A more direct approach, therefore, appears to be crosses between the two heterotic groups. Griffing (1963) and later on Cress (1966, 1967) already discussed the point that through reciprocal recurrent selection no improvement is possible on loci that are fixed in the two groups. They suggested to mix the two heterotic groups and from this population develop two new heterotic groups through continued reciprocal recurrent selection. This would be advantageous for all loci for which the unfavorable allele was fixed in one of the groups. The major drawback of such an approach, however, would be the reduction in the expected heterozygosity of hybrids and with it the reduced heterosis and hybrid performance after the mixing of the groups. Only in the long run would genetic distance between the new heterotic groups and thus the high levels of heterosis be restored. This radical approach, therefore, does not seem suitable for established hybrid breeding programs that need to stay competitive also in the short run. In addition, also in this scenario, loci would get fixed in the two groups through selection and random drift.

**Fig. 4:**
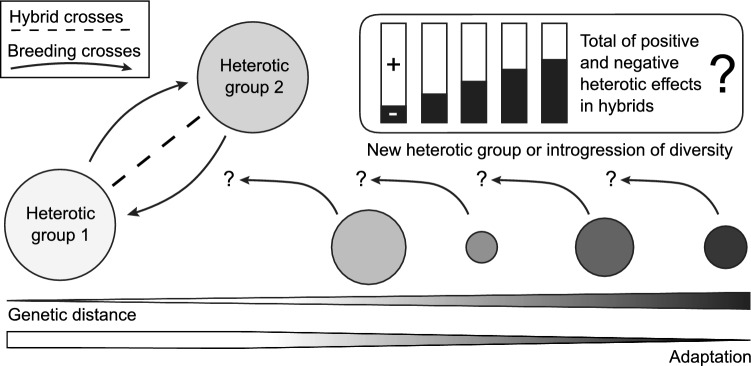
Schematic representation of the proposed organization of heterotic groups in hybrid breeding and the utilization of novel diversity. Shown are two heterotic groups that are improved by reciprocal recurrent selection, represented by the ‘Hybrid crosses’, which means that crosses between the groups are only made to evaluate the hybrid performance. In addition, ‘Breeding crosses’ might be made among them in order to relieve the fixation of alleles in these groups, meaning that crosses are made with the goal to develop novel lines from these crosses. Furthermore, different populations with increasing genetic distance to these two could be used either as a new heterotic group or to broaden the genetic diversity by introgression. It can be assumed that to a certain extent, the material becomes less adapted to the target environment the more genetically distant it is. The heterosis of this material with the used heterotic groups depends on the total of the positive and negative heterotic effects of each combination

We therefore propose an alternative approach. We suggest that breeding crosses are not only made within each heterotic group as done so far, but to a smaller extent also between them (Fig. 4). This gene flow between heterotic groups will release the fixation of alleles and will subsequently allow selection to become effective. Loci for which the fixation was favorable should not suffer much from this introgression and selection will work toward their re-fixation or near fixation. A potential limitation, at least in the short term, is coupling of heterotic loci in repulsion phase, as then the selection cannot act on single loci but only on the net effect of haploblocks comprising several loci. Importantly, these breeding crosses between the heterotic groups should be limited as this approach must balance two goals. One is the just mentioned exchange of alleles in order to break fixation, while at the same time the established heterotic pattern should not be disrupted. Notably, the approach, if effective, would not only reduce the genetic distance between groups but may in sum actually reduce heterosis. Eliminating negative heterotic effects will increase heterosis, while fixation of the favorable allele in both groups will remove positive dominance effects, thus reducing heterosis. Nevertheless, as the latter will simultaneously increase the midparent value, the approach would achieve the primary goal in hybrid breeding which is the maximization of the hybrid performance.

This approach is reminiscent of the breeding scheme called convergent improvement suggested in the early stages of hybrid breeding (Richey 1927). It is a double-backcross design in which the F_1_ of the cross A × B is backcrossed to both parents A and B. The idea was that if heterosis was due to dominance, it would be possible to exchange and accumulate favorable dominant alleles in the derived A‘ and B‘ lines, thereby improving them without affecting the hybrid performance. The motivation for this approach stemmed primarily from the poor performance of the early generation maize inbred lines and the necessity to improve them, while the idea of maintaining or increasing diversity was of no concern then. However, despite experiments supporting the approach, it was never widely adopted by maize breeders (Tracy and Chandler 2006). While the focus is now not so much the per se improvement of the lines any more but eliminating negative heterotic effects, the idea of crosses between the parental components of a hybrid, which nowadays means between heterotic groups, becomes attractive again. Consequently, a reduction in their genetic distance, though this may seem counter-intuitive or even sacrilegious to classical hybrid breeders, may be advantageous for hybrid breeding and the suggested approach deserves consideration.

### Consequences for the use of new material in hybrid breeding

Genebanks hold large numbers of accessions for all agriculturally relevant crops. These cover a vast space of genetic diversity and consequently include germplasm that maximizes the genetic distance to the material of any established or new hybrid breeding program. From this the question arises whether and if so, to what extent more exotic elite material or even genetic resources can be utilized to promote heterosis and with it hybrid performance. So how about new heterotic groups for an established hybrid breeding program or crops without established heterotic groups: how far distant should we go? As we have seen, we ideally maximize the genetic distance but only at heterotic loci with positive effect. In general, we probably have to expect more negative heterotic effects the more we maximize genetic distance, though this is likely substantially related to the issue of adaptation. So how to use distant elite material or even genetic resources for hybrid breeding?

The use of exotic material for monogenic traits is straightforward, as the target gene can simply be introgressed, ideally assisted by molecular markers to control the genetic background and the linkage drag. But what about quantitative traits and their potential to increase heterosis in hybrid breeding? If we ignore adaptation for the moment, this still leaves the question whether for traits like grain yield there are favorable alleles that can be contributed by a genetic resource. While elite material has been intensively selected, genetic resources have seen no or only little selection. This means that they will in most cases still harbor many alleles that are unfavorable and have therefore been purged from elite material through decades of selection. These loci will then be polymorphic between elite material and genetic resources, which will result in an increased genetic distance, that, assuming that genetic distance is favorable for heterosis, will make them appear attractive for hybrid breeding. Let us consider a genetic resource that has a large genetic distance to an established elite heterotic group but carries no favorable alleles at all. The effect on heterosis then depends on which of the two alleles is dominant. In the best case, only the elite alleles are dominant and contribute favorably to heterosis, even though that hybrid could only reach the performance of the elite line. In reality, however, it must be expected that of the exotic but unfavorable alleles, a portion that may vary between genetic resources, will be dominant and thus reduce heterosis. This illustrates that in addition to adaptation issues, genetic resources will in most cases not be suited to directly serve as a heterotic group in elite hybrid breeding. Another argument for this is, that it is not only heterosis that matters in a commercial hybrid, but ultimately the hybrid performance, which besides heterosis also depends on the midparent value and thus on the per se performance of the parental components. This is usually much lower in genetic resources and thus hybrids with them will rarely be competitive. Consequently, genetic resources can in most cases not be utilized directly as heterotic group but can serve to broaden the genetic basis of existing heterotic groups. An approach to mine the favorable alleles for complex traits that at least some genetic resources certainly contain, has been suggested by Longin and Reif (2014) and is based on performing GCA tests with the genetic resources.

So how to find a genetically distant group suitable to be used either as a heterotic group or for introgression of new diversity into already employed heterotic groups? For this, we need to consider not only heterosis, but mainly hybrid performance, and consequently the per se performance of the candidate material becomes important and with it its adaptation. The genetic distance to the established heterotic pattern can be easily determined by molecular markers. The mean performance and the adaptation to the target environment(s) can then be tested in field trials as a first step. The positive and negative heterotic effects can even with molecular markers not be determined beforehand, or only to a limited extent. This means that as a second step, the traditional approach of using testcrosses, so selected elite lines as testers or a tester mix from each of the established groups of a heterotic pattern, is used to produce hybrids with either the candidate population or if available with representative selected lines from that group. Based on the testcross performance, the decision is made whether a group is suited or not. In theory, one could as a next step extend the number of lines in the established target heterotic group and cross them with the candidate population or if inbred lines are available expand their number on both sides in order to use this population for mapping and effect estimation of heterotic loci, which could then be used for genomic-assisted approaches. However, the potential gain of such an approach will in most cases likely not justify the required resources and efforts.

Another question concerns crops without established heterotic groups, as for example wheat. Zhao et al. (2015) have recently presented an approach for the de novo grouping and prediction of a high-yielding heterotic pattern. From there on, the same applies as for crops with a long-standing hybrid breeding history and established heterotic patterns.

## Conclusions

In conclusion, hybrid breeding requires genetic distance between the parental components and thus between the heterotic groups in a breeding program. However, heterotic effects can be positive as well as negative and thus, heterosis and with it hybrid performance is not maximized by maximizing the genetic distance between parental components of a hybrid but by optimizing it.
